# The response of potato tuber yield, nitrogen uptake, soil nitrate nitrogen to different nitrogen rates in red soil

**DOI:** 10.1038/s41598-021-02086-5

**Published:** 2021-11-18

**Authors:** Kailou Liu, Jiangxue Du, Yijun Zhong, Zhe Shen, Xichu Yu

**Affiliations:** 1 Jiangxi Institute of Red Soil, National Engineering and Technology Research Center for Red Soil Improvement, Nanchang, 331717 People’s Republic of China; 2 Institute of Agricultural Resources and Regional Planning, Chinese Academy of Agricultural Sciences, Beijing, 100081 People’s Republic of China

**Keywords:** Biogeochemistry, Ecology, Environmental sciences

## Abstract

Nutrient-deficient red soil found in the southern region of China is increasingly being used for potato crops to meet the demand for this staple food. The application of nitrogen fertilizer is necessary to support the production of higher tuber yields; however, the links between nitrate nitrogen and the nitrogen balance in red soil are unknown. A field experiment was conducted in Jiangxi Province in 2017 and 2018 to determine the effects of different nitrogen application rates, 0 kg ha^−1^ (N0), 60 kg ha^−1^ (N60), 120 kg ha^−1^ (N120), 150 kg ha^−1^ (N150), 180 kg ha^−1^ (N180), 210 kg ha^−1^ (N210), and 240 kg ha^−1^ (N240, the highest rate used by local farmers), on potatoes growing in red soil. Data on tuber yield, crop nitrogen uptake, and the apparent nitrogen balance from the different treatments were collected when potatoes were harvested. Additionally, the content and stock of nitrate nitrogen at different soil depths were also measured. Nitrogen fertilization increased tuber yield but not significantly at application rates higher than 150 kg ha^−1^. We estimated that the threshold rates of nitrogen fertilizer application were 191 kg ha^−1^ in 2017 and 227 kg ha^−1^ in 2018, where the respective tuber yields were 19.7 and 20.4 t ha^−1^. Nitrogen uptake in potato in all nitrogen fertilization treatments was greater than that in N0 by 61.2–237% and 76.4–284% in 2017 and 2018, respectively. The apparent nitrogen surplus (the amount of nitrogen remaining from any nitrogen input minus nitrogen uptake) increased with increasing nitrogen application rates. The nitrate nitrogen stock at a soil depth of 0–60 cm was higher in the 210 and 240 kg ha^−1^ nitrogen rate treatments than in the other treatments. Moreover, double linear equations indicated that greater levels of nitrogen surplus increased the nitrate nitrogen content and stock in soils at 0–60 cm depths. Therefore, we estimate that the highest tuber yields of potato can be attained when 191–227 kg ha^−1^ nitrogen fertilizer is applied to red soil. Thus, the risk of nitrate nitrogen leaching from red soil increases exponentially when the apparent nitrogen balance rises above 94.3–100 kg ha^−1^.

## Introduction

As the fourth most important staple in the global food supply, potato (*Solanum tuberosum*) crops are grown in many countries^1^, such as China, India, Russia, Ukraine, and the United States of America. Currently, China is the largest potato producer in the world^2^. China’s potato crops are mainly planted in its western provinces, such as Gansu, Inner Mongolia, Ningxia, Sichuan and Yunnan^3^, as well as its southern region. However, the planting acreage is smaller and the tuber yield is lower in the southern region than in the western region^4^. The differences may be attributed to poor soil fertility, especially in red soils primarily located in the southern region^5^. Therefore, we aimed to improve potato production in the red soils of southern China through reasonable fertilizer management.

In red soil, nitrogen loss is high due to local rainfall patterns and soil erosion^6,7^. Many researchers have reported that more precise control of the total amount of applied nitrogen fertilizer could be an important measure for decreasing soil nitrogen loss^8,9^ and the apparent nitrogen balance (the positive or negative value calculated from nitrogen input minus nitrogen uptake). High nitrogen inputs will likely increase the apparent nitrogen balance of soils^10^ and result in greater accumulation of nitrate nitrogen in soil^11,12^ and thus higher nitrogen pollution risk. In the cultivation of major cereals (maize, wheat and rice), “there are large opportunities to reduce the environmental impact of agriculture by eliminating nutrient overuse (especially nitrogen), while still allowing an approximately 30% increase in production”^13^.

For potato production, the threshold rates of nitrogen fertilizer have been reported from studies conducted in the western region of China and other countries. For example, an average rate of 240 kg ha^−1^ nitrogen applied to fields in Northwest China^14^ and a lower rate of 200 kg ha^−1^ applied to fields in northern China^15^ achieved the highest yields of potato tubers. In the Columbia Basin in the Pacific Northwest of the United States, the optimum rate of nitrogen fertilizer for potato was 336 kg ha^−1^^11^. In Florida, U.S.A., Rens et al.^16^ determined that different application rates at different stages of potato growth achieved the highest tuber yield: 114–138 kg ha^−1^ nitrogen at emergence and 56 kg ha^−1^ nitrogen at tuber initiation. Moreover, the response of potato yield to nitrogen rate may differ due to the potato variety^17^. Reportedly, the tuber yield of heritage potato would be decreased under a nitrogen application rate greater than 80 kg ha^−1^ nitrogen, but the tuber yield of modern potato yield can be increased^18^. These potato studies did not investigate crops grown in red soil; however, determining ideal rates of nitrogen fertilizer application to red soil for achieving high yields of potato while reducing nitrogen pollution is needed. This is because nitrate nitrogen easily leaches from red soils due to abundant rainfall and high soil erosion, which elevates the importance of developing more sustainable fertilization strategies in fields with red soil.

A threshold rate of nitrogen fertilizer application to improve the nitrogen use efficiency of potatoes grown in red soils was the focus of our study. A field experiment testing nitrogen fertilizer rates and determining their effects on potato was conducted in red soil from 2017 to 2018. Yield, nitrogen uptake and apparent nitrogen balance data were analysed, and the relationship between the nitrate nitrogen stock and apparent nitrogen balance was discussed. Using the system of a potato crop grown in red soil, the objectives of this study were 1) to determine the threshold rate of nitrogen fertilizer application to achieve high tuber yield, 2) to investigate the changes in the nitrate nitrogen content and stock under different nitrogen fertilizer rates, and 3) to determine the relationship between the nitrate nitrogen stock and apparent nitrogen balance. Overall, the results will provide valuable empirical evidence to help improve potato production in red soils in China.

## Materials and methods

### Site description

The field experiment was performed in Xiaojiang Village in the town of Zhanggong, Jinxian County, Jiangxi Province, China (28° 35′ 24′′ N, 116° 17′ 60′′ E). This area has a typical subtropical climate with a mean annual precipitation of 1,727 mm and a mean annual temperature of 18.1 °C. The soil type is red soil according to the Chinese soil classification system, which is in the Plinthosol soil group according to the IUSS classification system^19^. The parent materials are quaternary red clay with kaolinite, the dominant mineral. The slope of the experimental field was 5°. Prior to conducting the experiment, the soil characteristics of the plough horizon (0–20 cm) were obtained and were as follows: the pH was 4.92; the soil organic carbon content was 11.3 g kg^−1^; and the total nitrogen and nitrate nitrogen contents were 0.95 g kg^−1^ and 8.55 mg kg^−1^, respectively.

### Experimental design

The field experiment was conducted in both 2017 and 2018. There were seven treatments, N0, N60, N120, N150, N180, N210, and N240, with increasing rates of nitrogen fertilizer: 0, 60 (the lowest rate used by local farmers), 120, 150, 180, 210, and 240 kg ha^−1^ (the highest rate used by local farmers), respectively. The seven treatments were randomized following a complete block design with three replicates. The plots were 30 m^2^ with a 6 m length and 5 m width.

Phosphorus (P_2_O_5_) and potassium (K_2_O) fertilizers were applied in the respective amounts of 65 and 116 kg ha^−1^ to all treatments. The kinds of nitrogen, phosphorus and potassium fertilizers were urea, calcium magnesium phosphate and potassium chloride, respectively. We added a mixture of 40% nitrogen, 100% phosphorus and 50% potassium fertilizer to the topsoil (0–20 cm) as basal fertilizer before planting potatoes. At the seedling stage (35 days after potato planting), 30% nitrogen fertilizer was spread onto the soil surface. At the flowering stage (60 days after potato planting), 30% nitrogen fertilizer and 50% potassium fertilizer were spread onto the soil surface.

The planting density was 4,200 plants ha^−1^, the sowing dates in 2017 and 2018 were the 22nd and 25th of February, and the harvest dates were the 18th and 22nd of May in 2017 and 2018. The row and plant spacing of potato were 30 cm and 20 cm, respectively. We used the ridge cultivation system to plant potatoes; ridges were 35 cm in height and spaced 60 cm apart.

All experiments were performed in accordance with relevant guidelines and regulations. Moreover, we obtained permission to collect potato plants from the Jiangxi Institute of Red Soil.

### Measurements

After harvesting plant materials in 2017 and 2018, potato tubers were manually excavated from each plot, collected and weighed to obtain tuber yield. Five plants were sampled from each plot, divided into tuber and straw, dried in an oven at 105 °C for 2 h, and then dried to a constant weight at 85 °C. The tubers and straw were pulverized by a micro plant grinding machine (model JFSO-480, Zhejiang Top Instrument Co., Ltd., Zhejiang Province, China) and then sieved manually through a 0.5-mm mesh.

The nitrogen contents of the plant samples were determined by the micro-Kjeldahl method^20^. Potato nitrogen uptake and the apparent nitrogen balance were calculated using the following equations:

1 $${N}_{uptake}={N}_{tuber}\times {Y}_{tuber}+{N}_{straw}\times {Y}_{straw}\times 0.001$$

2 $${N}_{balance}={N}_{input}-{N}_{uptake}$$

where ${{\varvec{N}}}_{{\varvec{u}}{\varvec{p}}{\varvec{t}}{\varvec{a}}{\varvec{k}}{\varvec{e}}}$ is nitrogen uptake (kg ha^−1^); ${{\varvec{N}}}_{{\varvec{t}}{\varvec{u}}{\varvec{b}}{\varvec{e}}{\varvec{r}}}$ and ${{\varvec{N}}}_{{\varvec{s}}{\varvec{t}}{\varvec{r}}{\varvec{a}}{\varvec{w}}}$ are the nitrogen contents in tuber and straw materials (g kg^−1^), respectively; ${{\varvec{Y}}}_{{\varvec{t}}{\varvec{u}}{\varvec{b}}{\varvec{e}}{\varvec{r}}}$ and ${{\varvec{Y}}}_{{\varvec{s}}{\varvec{t}}{\varvec{r}}{\varvec{a}}{\varvec{w}}}$ are the biomass yields of tubers and straw (kg ha^−1^), respectively; and ${{\varvec{N}}}_{{\varvec{b}}{\varvec{a}}{\varvec{l}}{\varvec{a}}{\varvec{n}}{\varvec{c}}{\varvec{e}}}$ and ${{\varvec{N}}}_{{\varvec{i}}{\varvec{n}}{\varvec{p}}{\varvec{u}}{\varvec{t}}}$ are apparent nitrogen balance and nitrogen fertilizer input (kg ha^−1^), respectively.

After plants were harvested in 2017 and 2018, soil samples at different depths (0–20, 20–40 and 40–60 cm) were collected. Five samples were collected from each plot using a shovel, and these samples were then mixed together to obtain one homogeneous sample per plot. In addition, three undisturbed soil cores (5 cm in diameter, 5.1 cm in height) were taken from each of the three soil depths of each treatment to determine the soil bulk density. Any visible roots, organic residues, and stone fragments were removed manually from all fresh samples.

The nitrate nitrogen in the soil was extracted with 2 M KCl and quantified using a continuous flow injection analyser (model: Proxima, ALLIANCE Company, France). Nitrate nitrogen stocks at the different soil depths were calculated as follows: the nitrogen contents of the plant samples were determined by the micro-Kjeldahl method^20^. Potato nitrogen uptake and the apparent nitrogen balance were calculated using the following equations:

3 $${NNS}_{0-20}={NNC}_{0-20}\times {BD}_{0-20}\times 0.2\times 0.1$$

4 $${NNS}_{20-40}={NNC}_{20-40}\times {BD}_{20-40}\times 0.2\times 0.1$$

5 $${NNS}_{40-60}={NNC}_{40-60}\times {BD}_{40-60}\times 0.2\times 0.1$$

6 $${NNS}_{0-60}={NNS}_{0-20}+{NNS}_{20-40}+{NNS}_{40-60}$$

where ${{\varvec{N}}{\varvec{N}}{\varvec{S}}}_{0-20}$, ${{\varvec{N}}{\varvec{N}}{\varvec{S}}}_{20-40}$, and ${{\varvec{N}}{\varvec{N}}{\varvec{S}}}_{40-60}$ are the nitrate nitrogen stocks (kg ha^−1^) of the 0–20-, 20–40-, and 40–60-cm soil samples, respectively. ${{\varvec{N}}{\varvec{N}}{\varvec{C}}}_{0-20}$, ${{\varvec{N}}{\varvec{N}}{\varvec{C}}}_{20-40}$**,** and ${{\varvec{N}}{\varvec{N}}{\varvec{C}}}_{40-60}$ are the nitrate-nitrogen contents (mg kg^−1^) of the 0–20-, 20–40- and 40–60-cm soil samples, respectively. ${{\varvec{B}}{\varvec{D}}}_{0-20}$, ${{\varvec{B}}{\varvec{D}}}_{20-40}$**,** and ${{\varvec{B}}{\varvec{D}}}_{40-60}$ are the respective bulk density (g cm^−3^) values of the same three soil samples. In addition, ${{\varvec{N}}{\varvec{N}}{\varvec{S}}}_{0-60}$ is the nitrate nitrogen stock (kg ha^−1^) of the total soil depth of 0–60 cm, which was the sum of ${{\varvec{N}}{\varvec{N}}{\varvec{S}}}_{0-20}$, ${{\varvec{N}}{\varvec{N}}{\varvec{S}}}_{20-40}$, and ${{\varvec{N}}{\varvec{N}}{\varvec{S}}}_{40-60}$.

### Data analysis

Analysis of variance was performed using SPSS 19 software. The least significant difference (LSD at P < 0.05) test was applied to assess the differences in means (n = 3) among treatments. The relationship between tuber yield and nitrogen rate was fitted by a parabolic equation. The relationships between nitrate nitrogen stocks at different soil depths (0–20, 20–40 and 40–60 cm) and the apparent nitrogen balance were fitted by exponential equations. Last, the relationship between the nitrate nitrogen stock at the 0–60-cm soil depth and the apparent nitrogen balance was fitted by double linear equations. All figures were generated using Origin 8.5.

## Results

### Tuber yield

**Figure 1:**
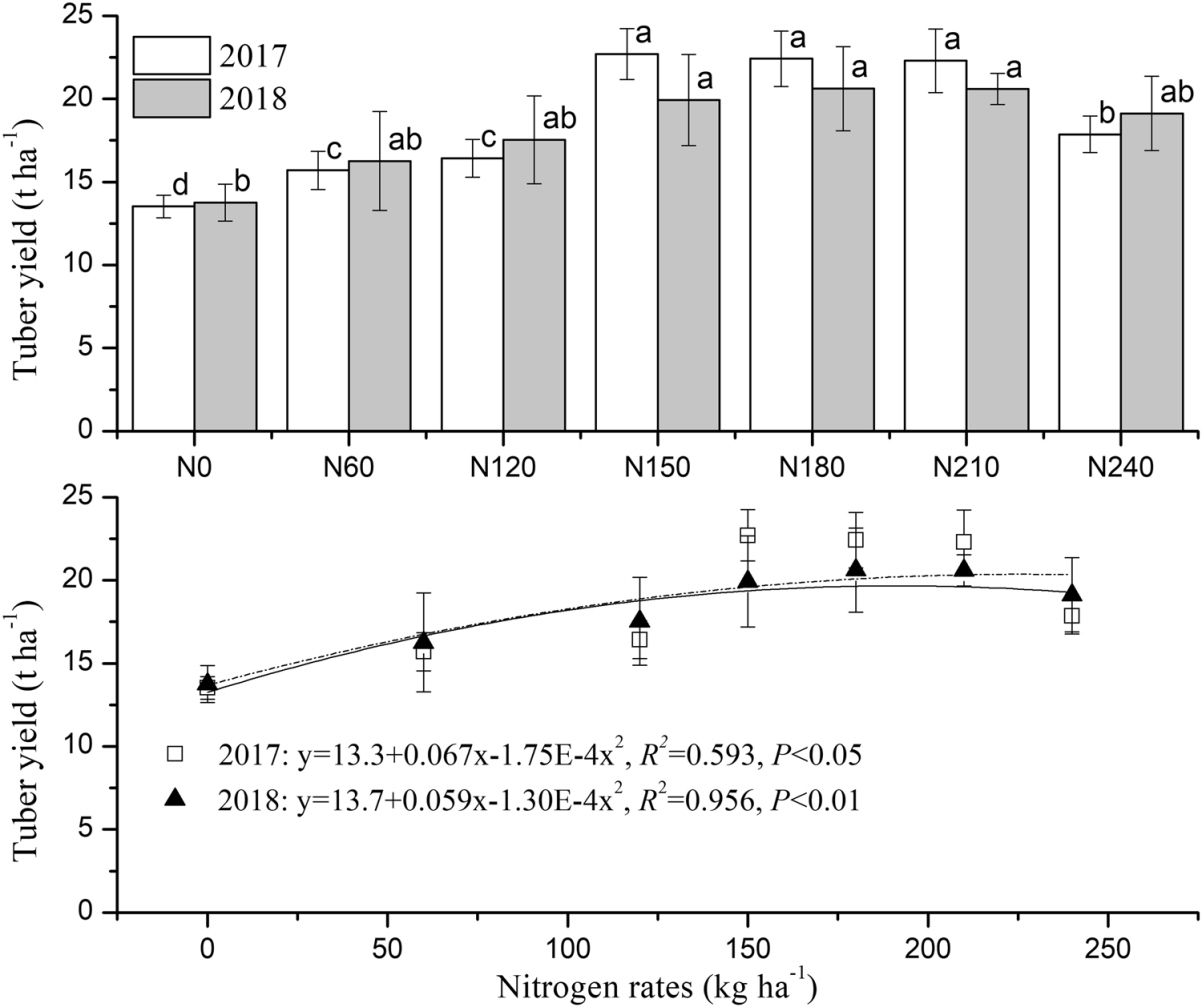
Potato tuber yield in treatments with different nitrogen fertilizer rates. Different letters indicate significant differences (P < 0.05) among treatments in the same year.

Potato tuber yield increased gradually under 0 to 150 kg ha^−1^ of applied nitrogen (Fig. 1). Compared with the yield in N0, the yields in N60, N120 and N150 were greater by 16.1%, 21.5% and 67.9%, respectively, in 2017 and 18.2%, 27.4% and 44.9%, respectively, in 2018. However, at nitrogen rates of more than 150 kg ha^−1^, yield did not significantly differ. Furthermore, the fitted parabolic equation of each dataset from 2017 and 2018 showed maximum tuber yields of 19.7 and 20.4 t ha^−1^, respectively, where the nitrogen rates were 191 and 227 kg ha^−1^, respectively.

### Potato nitrogen uptake

Nitrogen uptake in potato differed among all the treatments (Table 1). In both 2017 and 2018, the highest nitrogen uptake was attained in the N240 treatment, and the lowest uptake was attained in the N0 treatment. Compared with that in N0, the uptake levels measured in the N60, N120, N150, N180, N210 and N240 treatments were greater by 61.2%, 100%, 115%, 144%, 142% and 237%, respectively, in 2017. Meanwhile, the uptake levels measured in the N60, N120, N150, N180, N210 and N240 treatments were greater than that in the N0 treatment by 76.4%, 98.5%, 142%, 198%, 190% and 284%, respectively, for 2018. There were no significant differences among the N120, N150, N180 and N210 treatments in 2017. There were also no significant differences among the N150, N180 and N210 treatments in 2018.

**Table 1:** Nitrogen uptake in potato applied with treatments of different nitrogen fertilizer rates.

Treatments	Nitrogen uptake (kg ha^−1^)	
	2017	2018
N0	18.6 ± 3.58d	18.0 ± 3.04d
N60	30.0 ± 6.15c	31.8 ± 7.86c
N120	37.2 ± 5.71b	35.7 ± 5.03c
N150	39.9 ± 3.72b	43.7 ± 3.81b
N180	45.4 ± 6.06b	53.7 ± 4.04ab
N210	44.9 ± 1.79b	52.3 ± 4.56ab
N240	62.7 ± 5.42a	69.1 ± 5.06a

Different letters indicate significant differences (P < 0.05) among treatments in the same year.

### Apparent nitrogen balance

**Figure 2:**
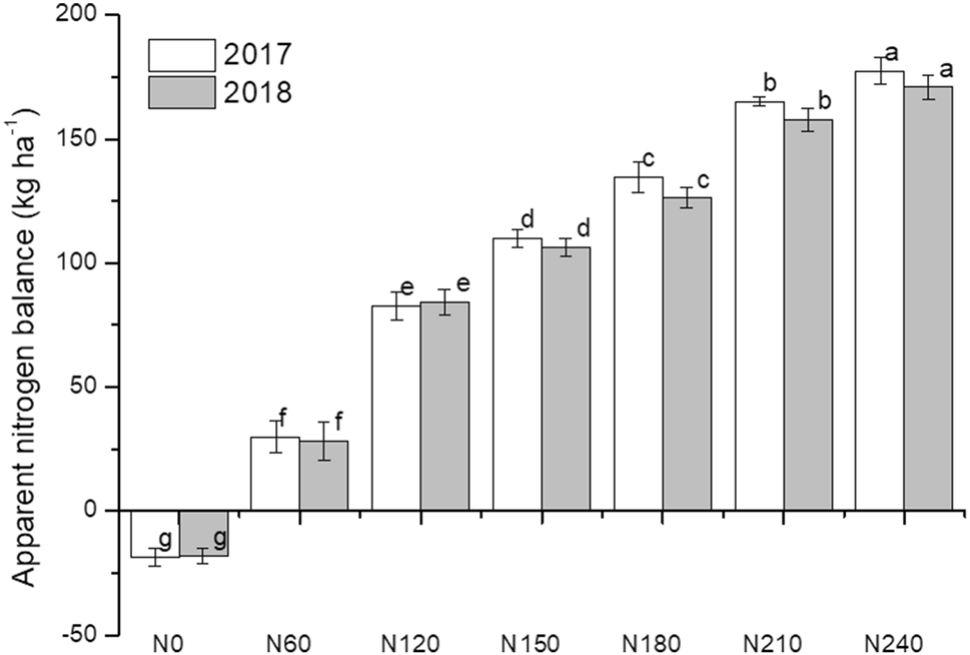
Apparent nitrogen balance (amount of nitrogen input minus amount of plant nitrogen uptake) calculated for potato treated with different nitrogen fertilizer rates. Different letters indicate significant differences (P < 0.05) among treatments in the same year.

The apparent nitrogen balance in potato production in red soil increased with increasing nitrogen application rates (Fig. 2). For both 2017 and 2018, all treatments with nitrogen application exhibited a surplus of nitrogen, while the N0 treatment exhibited a deficiency of nitrogen (18.6 and 18.0 kg ha^−1^ in 2017 and 2018, respectively). Furthermore, the surplus was highest in the N240 treatment. Compared with that in the N60 treatment, the nitrogen surplus levels in the N120, N150, N180, N210 and N240 treatments were 176%, 266%, 348%, 450% and 490% greater in 2017 and 198%, 277%, 347%, 459% and 505% greater in 2018, respectively.

### Nitrate nitrogen content and stock at different soil depths

**Figure 3:**
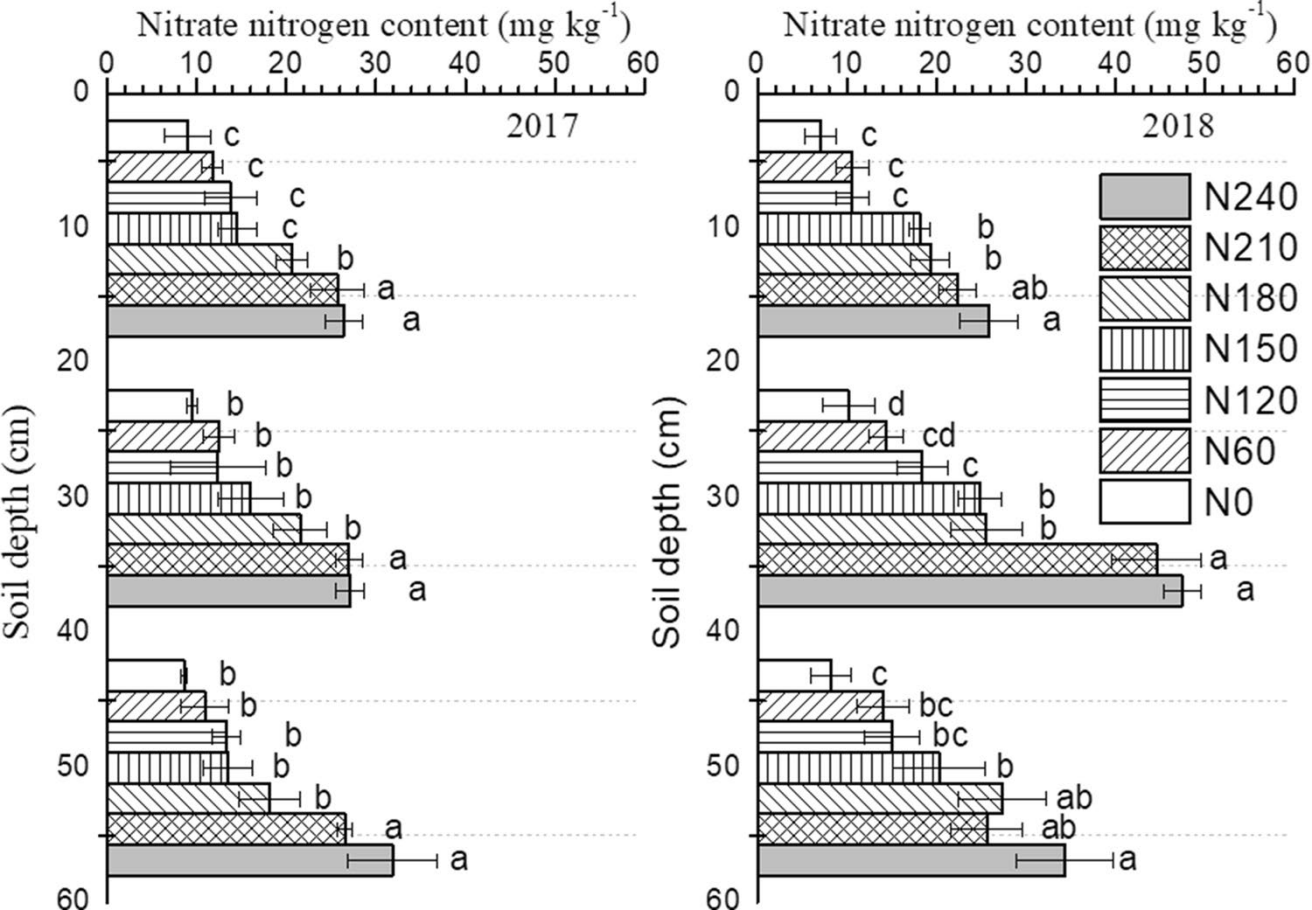
Nitrate nitrogen contents at different soil depths in treatments with different nitrogen fertilizer rates. Different letters in the same soil depth indicate significant differences (P < 0.05) among treatments in the same year. Nitrogen balance (amount of nitrogen input minus amount of plant nitrogen uptake) calculated for potato treated with different nitrogen fertilizer rates.

Nitrogen application increased the nitrate nitrogen content at all three depths of soil samples (Fig. 3). Generally, as nitrogen fertilizer rates increased, the nitrate nitrogen content increased in both 2017 and 2018 samples. For the same treatments among the three depths of soil in 2017, there were no significant differences in nitrate nitrogen contents. The result for 2018 was similar to that for 2017, with the exceptions of the N210 and N240 treatments, both of which showed higher nitrate nitrogen contents at 20–40 cm than at 0–20 and 20–40 cm. The nitrate nitrogen contents in N210 and N240 were higher than the contents in the other treatments among the three soil depths. Compared with the content in the N0 treatment, the nitrate nitrogen contents at 0–20 cm in the N210 and N240 treatments were 185% and 193% greater in 2017 and 218% and 268% greater in 2018, respectively. The nitrate nitrogen contents at 20–40 cm in the N210 and N240 treatments were 186% and 188% greater in 2017 and 339% and 367% greater in 2018, respectively. The nitrate nitrogen contents at 40–60 cm in the N210 and N240 treatments were 209% and 271% higher in 2017 and 212% and 318% higher in 2018, respectively.

There was less consistency in significant differences in nitrate nitrogen content among treatments with less than 210 kg ha^−1^ nitrogen fertilizer applied. In the 2017 samples, no significant differences were observed among N0, N60, N120, N150 and N180, with the exception of the nitrate nitrogen content in N180 being higher than those in N0, N60, N120, and N150 at the 0–20-cm soil depth. In the 2018 samples, the nitrate nitrogen contents in N180 and N150 were higher than those in N0, N60, and N120 at the 0–20- and 20–40-cm soil depths. In contrast, the nitrate nitrogen content in the N180 treatment at the 40–60-cm soil depth was not lower than that in the N240 and N210 treatments. Additionally, no significant differences were observed at the 40–60-cm depth among the N0, N60, and N120 treatments, but the nitrate nitrogen contents of the N150 and N180 treatments were higher than the content of the N0 treatment by 57.3% and 111% in 2017 and by 147% and 234% in 2018, respectively.

The nitrate nitrogen stock results were similar to the nitrate nitrogen content results. Table 2 shows that there were no significant differences in nitrate nitrogen stock among the three depths of soil in 2017. In 2018, the nitrate nitrogen stocks at 20–40 cm were higher than those at 0–20 and 20–40 cm. Compared with those in 2017, the nitrate nitrogen stocks at 0–60 cm (sum of measurements from the three depths) in 2018 were greater due to the application of nitrogen fertilizer. The nitrate nitrogen stock was higher in the nitrogen application treatments among the three soil depths than in the no-nitrogen fertilizer treatment (Table 2). A general trend of higher levels was also observed in the N210 and N240 treatments compared with the other treatments. The nitrate nitrogen stock at 0–60 cm was greater in the N210 and N240 treatments than in the N180 treatment by 31.6% and 41.6% in 2017, respectively, and 30.5% and 51.6% in 2018, respectively. For the three soil depths in 2017, there were no significant differences among the N0, N60, N120 and N150 treatments, but their nitrogen stocks were lower than that of the N180 treatment (except for that at the 40–60 cm depth). The results in 2018 revealed no significant differences among the N0, N60 and N120 treatments, but their nitrogen stocks were all lower than those in the N150 and N180 treatments.

**Table 2:** Nitrate nitrogen stock at different soil depths in treatments with different nitrogen fertilizer rates.

Year	Treatments	Nitrate nitrogen stock at different soil depths (kg ha-1)			
		0–20-cm	20–40-cm	40–60-cm	0–60-cm
2017	N0	29.0 ± 8.19c	27.8 ± 1.84c	21.8 ± 0.91c	78.7 ± 11.0c
	N60	35.7 ± 3.72c	36.2 ± 4.90c	26.4 ± 6.58c	98.2 ± 15.2c
	N120	43.6 ± 9.32c	36.3 ± 15.7c	33.4 ± 3.79c	113 ± 28.8c
	N150	45.6 ± 6.82c	46.8 ± 10.7bc	33.8 ± 6.85c	126 ± 24.4c
	N180	63.1 ± 5.39b	62.9 ± 8.73b	44.6 ± 8.55c	171 ± 22.7b
	N210	80.2 ± 9.33a	78.4 ± 4.36a	65.9 ± 2.04b	224 ± 15.7a
	N240	82.4 ± 6.35a	79.4 ± 4.68a	79.7 ± 12.5a	241 ± 23.5a
2018	N0	22.6 ± 5.55c	29.9 ± 8.61c	20.9 ± 5.84c	73.4 ± 20.0c
	N60	32.2 ± 5.65c	41.6 ± 5.39c	33.9 ± 7.00bc	108 ± 18.0c
	N120	33.4 ± 5.83c	54.2 ± 8.34c	37.6 ± 7.78bc	125 ± 22.0c
	N150	56.9 ± 3.59b	72.6 ± 6.96b	50.7 ± 12.9b	180 ± 23.4b
	N180	59.2 ± 6.67b	74.7 ± 11.5b	67.5 ± 12.1ab	201 ± 30.3b
	N210	69.8 ± 6.45ab	129 ± 14.7a	63.6 ± 9.84ab	263 ± 31.0a
	N240	80.7 ± 9.90a	139 ± 6.13a	86.0 ± 13.5a	305 ± 29.6a

Different letters at the same soil depth indicate significant differences (P < 0.05) among treatments in the same year.

### The relationship between the apparent nitrogen balance and nitrate nitrogen stock

**Figure 4:**
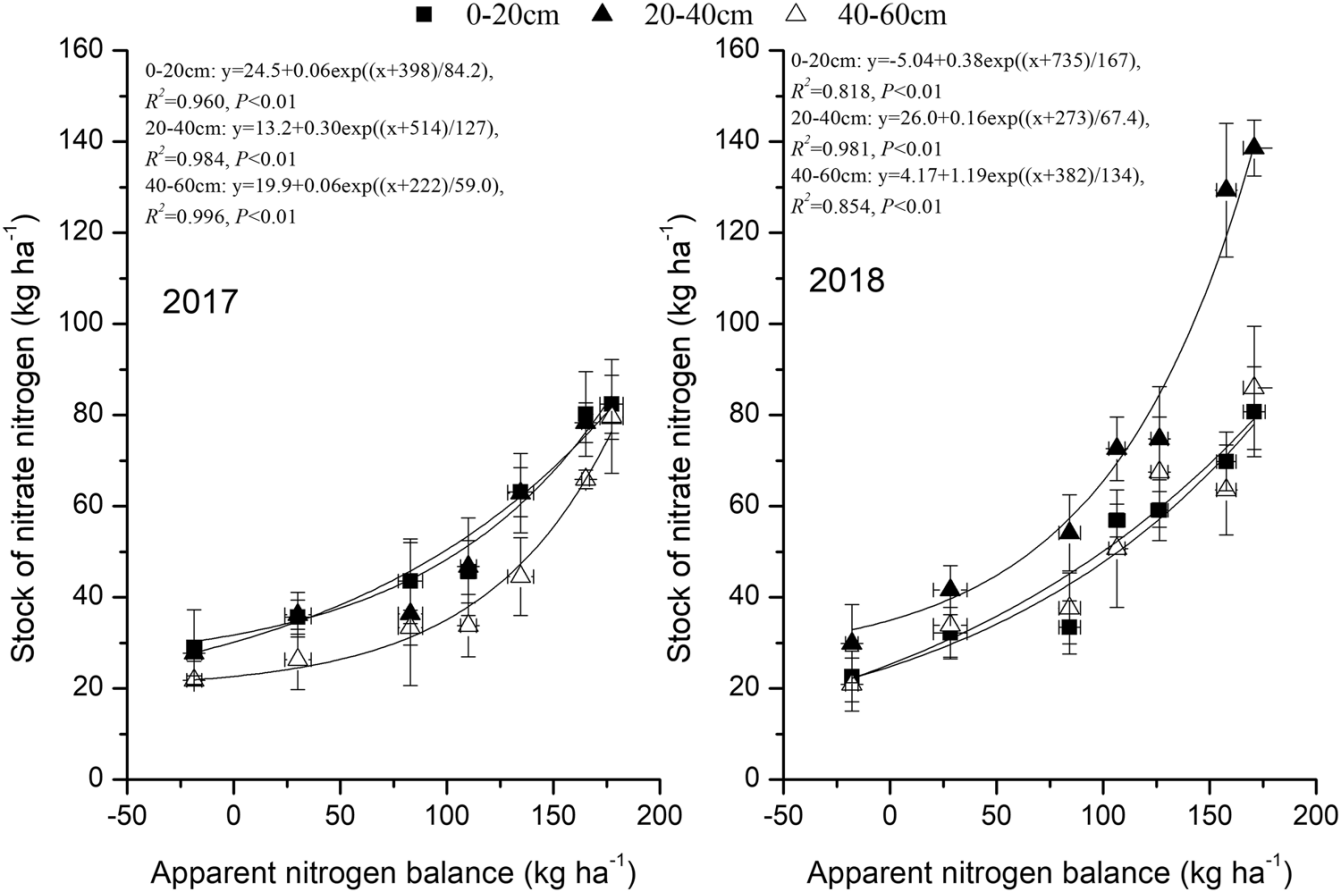
The relationship between the apparent nitrogen balance and nitrate nitrogen stock at different soil depths.

The nitrate nitrogen stock at the 0–20-, 20–40- and 40–60-cm soil depths increased exponentially with the apparent nitrogen balance (Fig. 4). The relationship at each of the three depths showed a gradual rate of increase in the nitrate nitrogen stock as the values calculated for the apparent nitrogen balance increased.

**Figure 5:**
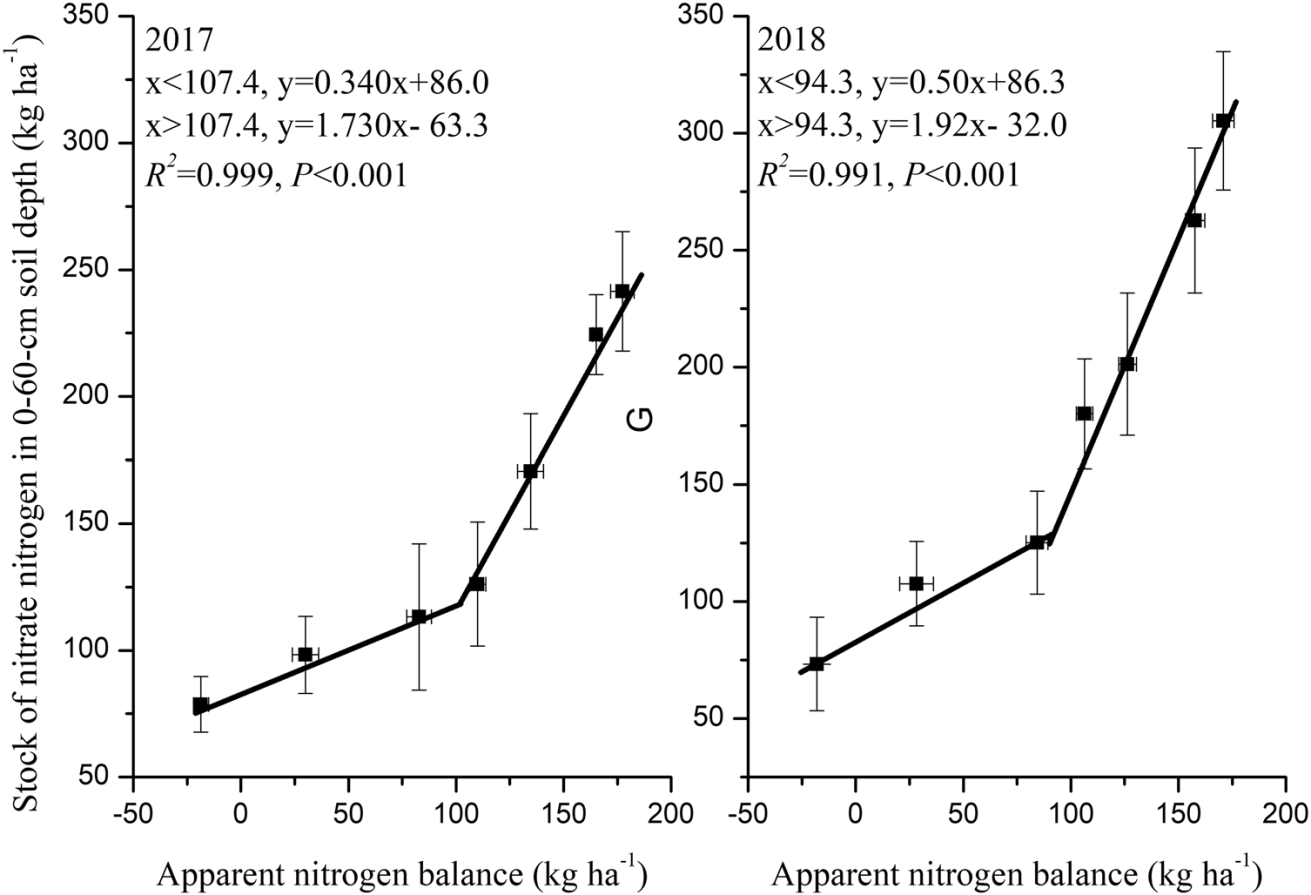
The relationship between the apparent nitrogen balance and nitrate nitrogen stock at the total soil depth of 0–60 cm.

The relationship between the apparent nitrogen balance and nitrate nitrogen stock at the 0–60-cm soil depth was fitted by double linear equations in both 2017 and 2018 (P < 0.01, Fig. 5). The rates of increase in nitrate nitrogen stocks in 2017 and 2018 were 0.34 and 0.50 kg ha^−1^, respectively, as the apparent nitrogen balance increased up to 100 and 94.3 kg ha^−1^, respectively. However, beyond those values of the apparent nitrogen balance, the slopes of the equations were steeper, indicating faster rates of increase for 2017 and 2018 at 1.73 and 1.92 kg ha^−1^, respectively.

## Discussion

### Tuber yield, nitrogen uptake and apparent nitrogen balance increased with increasing rates of nitrogen application to red soil

In general, the natural supply of nitrogen in red soil is inadequate to support the production of crops^21^, so the application of nitrogen fertilizer has been necessary to meet the demands for higher crop yields^22^. Unlike in the western region of China, a threshold rate of nitrogen fertilizer application has yet to be determined for potato planting in red soil^2–4^. In this study, potato yield increased with increasing amounts of applied nitrogen fertilizer. This result was similar to other findings^11,14,16,18^. In addition, we estimated that maximum tuber yields of 19.7 and 20.4 t ha^−1^ could be attained with nitrogen application rates to red soil of 191–227 kg ha^−1^. However, our tuber yields were lower than those observed in other regions^11,15,16,18^; for example, at the provincial level in China, Wang et al.^15^ used two crop growth models and estimated potato yields to vary from 38.8 t ha^−1^ to 66.4 t ha^−1^ in 2006–2015. In addition to the potential effect of crop varieties, the likely reason for our lower yield estimates in red soil is the inherent lower soil fertility^23^ than that observed in the western region of China. Additionally, the higher proportion of clay in red soil^23,24^, which indicates poor soil structure, likely suppressed tuber enlargement of the potatoes grown in that type of soil. Potatoes grow better in soils with less clay and a more developed soil structure^25^. A second possible factor affecting yield is the rainfall that occurs in spring, when potato vegetative growth primarily occurs. More rainfall suggests a greater presence of clouds that reduce the amount of sunlight reaching potato leaves, resulting in reduced photosynthetic activity and in turn limiting tuber production to meet higher yields. However, for the red soil region, there were different rainfall patterns and air temperatures at different sites, and their soil fertility levels varied. Therefore, the threshold rate of nitrogen application for higher potato tuber yields in this study will be further verified at other sites.

Other nutrient deficiencies can affect nitrogen deficiency in soil. Grzebisz et al.^26^ reported that increased amounts of potassium in tubers at harvest might be a crucial factor for controlling the partial factor productivity of fertilizer nitrogen, subsequently decreasing nitrogen rates. Red soil is potassium deficient^27^; therefore, we suggest that application rates of potassium fertilizer should also be optimized towards specific production goals.

In the red soil, nitrogen uptake in potato increased gradually with increasing nitrogen application rates (0–240 kg ha^−1^) to the soil. However, the nitrogen uptake by potato in our study was lower than that reported for potato grown in the western region of China and was likely due to the lower biomass observed in our study^4^.

Higher inputs of nitrogen fertilizer can lead to a nitrogen surplus in red soil, although some nitrogen may be removed by severe soil erosion resulting from rainfall^6,14^. We found that the amount of nitrogen surplus was more than 25 kg ha^−1^ among all nitrogen fertilization treatments. Moreover, the amounts were much higher, more than 150 kg ha^−1^, in the treatments with the highest nitrogen rates, 210 and 240 kg ha^−1^. However, the amounts of nitrogen surplus differed from those at other sites reported in other studies, and the differences were likely due to soil type, type and variety of crop, and other factors. Because a higher nitrogen surplus increases nitrogen pollution risk via runoff and leachates^28^, to reduce the risk from the excess nitrogen in the soil, we recommend a rotation of another crop, such as maize, in the same fields after the potatoes are harvested.

### Nitrate nitrogen content and stock increased with increasing nitrogen rates applied to red soil

The ridge cultivation system is commonly used in potato cultivation^14^. Because of this system, most of the applied nitrogen fertilizer was likely mixed into the soil to a depth of approximately 55 cm; the ridge height was approximately 35 cm plus a 20-cm tillage layer. Our results illustrated that there were no significant differences in nitrate nitrogen contents and stocks among the three depths of soil in 2017, indicating that the nitrogen fertilizer was likely well homogenized to at least a 60 cm soil depth. In the 2018 samples, the nitrate nitrogen contents at the 20–40 cm depth in the N210 and N240 treatments were higher than those at the 0–20 and 20–40 cm depths. The nitrate nitrogen stocks at 20–40 cm in all treatments were higher than those at 0–20 cm and 20–40 cm. We attribute these patterns in stock amounts and soil depth to the fact that potatoes were planted at a depth of approximately 30 cm in the ridge cultivation system, and all the nitrate nitrogen from the urea fertilizer was likely absorbed by the roots, moving the nitrogen to the 30-cm depth of soil^29^.

Soil nitrate nitrogen availability prior to the application of nitrogen fertilizer is also an important factor affecting plant nitrogen use efficiency^30^. In a novel aeroponics system, Tiwari et al.^31^ found that plant nitrogen availability was greater under controlled conditions with high nitrogen (7.5 mmol L^−1^ nitrate nitrogen) levels than under those with low nitrogen (0.75 mmol L^−1^ nitrate nitrogen) levels. Between the two years of soil sampling, the 2018 samples contained higher nitrate nitrogen stocks at 0–60 cm in all nitrogen fertilization treatments than the 2017 samples. The higher level in the second year was probably due to residual nitrogen fertilizer unused from the previous year^32^. After nitrogen fertilization application, some of the nitrogen will be absorbed by the crop, and the rest of the applied nitrogen may stay in a reserve at 0–60-cm soil depth in the form of nitrate nitrogen, likely from the applied urea.

Our data showed that the nitrate nitrogen content and stock increased significantly with increasing nitrogen application rates. Our result was consistent with that of Woli et al.^11^. Using a meta-analysis, Cui et al.^33^ also found that both reactive nitrogen losses and total N_2_O emissions from nitrogen fertilization increased exponentially with an increasing nitrogen application rate. However, the more nitrate nitrogen unabsorbed by roots and found in the soil, the more nitrogen may leach from the soil and pollute groundwater^34^.

### The response of nitrate nitrogen stocks to the apparent nitrogen balance of potato under different nitrogen application rates

Our results suggest a positive exponential relationship between nitrogen surplus and the soil nitrate nitrogen stock, as indicated by the gradual increase in the nitrate nitrogen stock with the increase in the apparent nitrogen balance. Moreover, the double linear equations of this relationship suggest that the turning point from a gradual to a steep increase in nitrogen surplus levels occurred at an apparent nitrogen balance of approximately 94.3–100 kg ha^−1^. The rate of increase in the nitrate nitrogen stock was 1.73–1.92 kg ha^−1^ at the total soil depth of 0–60 cm. When the nitrogen surplus rose above 94.3–100 kg ha^−1^, the stock was higher than that (0.34–0.50 kg ha^−1^) when the nitrogen surplus was less than 94.3–100 kg ha^−1^. This result was consistent with the relationship between soil nitrate nitrogen levels and potato yield reported by Nurmanov et al.^30^. In the dark chestnut heavy loamy soils of central Kazakhstan, Nurmanov et al.^30^ found that the maximum possible potato yield was achieved when soil nitrate nitrogen was lower than 22 mg kg^−1^^30^. Therefore, the 94.3–100 kg ha^−1^ range of nitrogen surplus could be used as a point of reference for soil nitrate nitrogen management and nitrogen fertilization strategies aiming to the threshold rate of fertilizer application rates for potato production in red soil.

## Conclusions

The threshold rate of nitrogen fertilizer applied to red soil for attaining high tuber yields in potato was estimated to be between 191 and 227 kg ha^−1^. There was no significant increase in yield above 150 kg ha^−1^ applied nitrogen fertilizer. The nitrogen balance passed a threshold of 100 kg ha^−1^ at nitrogen fertilizer application rates above 150 kg ha^−1^. A positive relationship between the apparent nitrogen balance and nitrogen application rate was observed. Above that threshold, the nitrate stock remaining in the soil increased dramatically, increasing the potential for nitrogen loss. For sustainable production in red soils, the rate of nitrogen fertilizer application should not exceed 150 kg ha^−1^. Therefore, this research will be helpful for the sustainable productivity of potato in red soil.

## Data Availability

Data reported within the article.
